# The Application of High-Frequency Ultrasonography in Post-Therapeutic Assessment of Actinic Keratosis After Photodynamic Therapy

**DOI:** 10.3390/cancers16223778

**Published:** 2024-11-09

**Authors:** Katarzyna Korecka, Anna Slian, Joanna Czajkowska, Aleksandra Dańczak-Pazdrowska, Adriana Polańska

**Affiliations:** 1 Department of Dermatology, Poznan University of Medical Sciences, 61-701 Poznań, Poland; aleksandra.danczak-pazdrowska@ump.edu.pl (A.D.-P.); apolanska@ump.edu.pl (A.P.); 2 Department of Medical Informatics and Artificial Intelligence, Silesian University of Technology, 41-800 Zabrze, Poland; anna.slian@polsl.pl

**Keywords:** actinic keratosis, high-frequency ultrasonography, photodynamic therapy

## Abstract

Objectives: Actinic keratoses (AKs) are one of the most common reasons for consultation in the elderly population. This study aimed to assess the efficacy of 5-ALA PDT in AK treatment using high-frequency ultrasonography (HFUS) to evaluate skin layer changes during therapy. Methods: In our study, we included 44 AK patients aged 53 to 89 years. All patients had lesions clinically evaluated with the Olsen and AKASI scale. HFUS imaging was performed on seemingly healthy skin and lesions before and at 4, 8, and 12 weeks of therapy. Ultrasound markers such as skin thickness, echogenicity, and pixel intensity were measured. 5-ALA was applied under occlusion for 3 h. After removing the occlusive dressing, 5-ALA was removed with a saline solution and a directed therapy with a BF-200 lamp. Full follow-ups of 56 markers of suitable quality were selected. Results: The thickness of SLEB significantly decreased in the following weeks compared to the pre-therapy results, reaching its lowest values after 12 weeks. The average pixel intensity significantly increased in each skin layer after therapy (p < 0.01). For SLEB, there were statistically significant differences in LEP, MEP and contrast. The AKASI score before and after treatment was determined for the 39 patients who underwent follow-up at week 12. The median AKASI score was 3.2 (1.2–8.6) before treatment and 0.6 (0–2.8) after. Conclusions: According to the literature data, this is the first study describing the ALA-PDT treatment efficacy in different AK severities evaluated in HFUS. HFUS provides a valuable non-invasive tool for monitoring the efficacy of PDT in AK treatment, showing significant improvements in skin texture and structure.

## Simple Summary

Actinic keratosis (AK) is one of the most frequent reason for consultations in dermatological offices. Many treatment options are available, one of them being photodynamic therapy (PDT), an in-office method with high treatment efficacy and acceptable cosmetic effects. This study aimed to evaluate the changes observed in a non-invasive skin imaging method—highfrequency ultrasonography (HFUS)—after PDT. We observed the decrease in SLEB after therapy and showed that this parameter maybe useful in monitoring the effects of treatment and, if not reduced completely, possibly indicating a potential risk of relapse. Additionally, for the first time, we propose the use of new USG parameters in this setting, i.e., LEP, HEP, MEP, homogeneity, and EPI, which present the possibility of overall assessment of patients after PDT, taking into account skin analysis at all levels. Our results show the improvement in skin texture mirrored in the analyzed parameters corresponding to the clinical pictures.

## 1. Introduction

Actinic keratoses (AKs) are among the most common reasons for consultation in the elderly population [1]. They are associated with extensive, cumulative sun exposure, predominantly in phototypes I-II on the Fitzpatrick scale [2,3]. Ultraviolet (UV) radiation affects the cells, leading to oxidative stress, and impacts tumor suppression protein, especially p53 [4], which might contribute to the progression of Squamous Cell Carcinoma (SCC). Risk factors that may increase the transformation to malignancy are immunosuppression, iatrogenic, and non-iatrogenic factors (such as in patients with Hodgkin’s lymphoma or chronic leukemia). Therefore, this group of patients should be under regular follow-up [5]. The progression model to SCC has many different endo- and exogenous factors. Genetically, TP53 and KNSTRN genetic mutations might be involved. The involvement of HPV in this process remains controversial [6].

Clinically, AKs present as red, scaly, well-circumscribed lesions on the scalp, face (the most frequent), and extremities [7]. Usually, they are graded according to the Olsen scale, which is based on an assessment of the thickness of the lesion and the presence of scales. Lesions classified as grade 1 are invisible but palpable; in grade 2, they are visible and palpable; in grade 3, they are very thick and hyperkeratotic [8]. In recent years, many non-invasive skin imaging techniques have allowed for a precise diagnosis of AKs without unnecessary biopsies. The application of dermatoscopy or reflectance confocal microscopy allows for the determination of an accurate diagnosis before the treatment [9,10].

High-frequency ultrasonography (HFUS) is a widely used, non-invasive method used in dermatology for many years. Higher frequencies (18–20 Mhz) allow the visualization of tumor infiltration before surgical excision, especially in melanoma or basal cell carcinoma [11,12,13], while the applications of this method in AKs are limited. The tumors usually manifest as anechoic or hypoechoic oval structures, with the possibility of subepidermal low-echogenic band formation underneath the entry echo (known as SLEB). This parameter also allows for the monitoring of treatment efficacy in some other dermatologic entities such as psoriasis, mycosis fungoides, or atopic dermatitis [14,15,16]. As dermatoscopy is a widely applied method that allows fast, non-invasive evaluations of suspicious lesions, HFUS enables the visualization of deeper layers of the skin. A comparison between dermatoscopy and HFUS is featured in Table 1.

The treatment modalities for AKs start with proper photoprotection, such as sunscreens and protective clothing. However, they are usually combined with various therapeutic methods. Many available options feature in-office or out-of-office settings. The treatment efficacy varies between methods and depends on the number of lesions, age, and patient compliance [1]. It is usually assessed with an AKASI score, which objectively allows the monitoring of treatment outcomes with different modalities [17].

Currently available therapeutics include 5-fluorouracil cream, cryosurgery, curretage, shave or surgical excisions, diclofenac 3% gel, imiquimod, photodynamic therapy (ALA-PDT and MAL-PDT), and the newest method, tirbanibulin [6,18]. The main aim is to prevent progression to SCC. Since the clinical presentation or thickness does not predict the risk of transformation, prompt treatment is recommended [19].

One currently recommended option is photodynamic therapy (PDT), an in-office method that relies on applying a photosensitizer under occlusion. Usually, 5-aminolevulinic acid (5-ALA) or methyl-aminolevulinic (MAL) acid are used. After the occlusion is removed, the lesions might be evaluated with ultraviolet-induced fluorescence dermatoscopy to assess the fluorescence due to the presence of Protoporphyrin IX [20]. Then, the lesions are exposed to red (630 nm) or blue (417 nm) light [21,22]. A daylight option may also be chosen if weather conditions allow [23].

So far, there is a little information on the application of HFUS in AK—there is only one study assessing its features with a 22–50 MHz transducer in 54 lesions. The most commonly described features for AKs have been the irregular basal border of the lesion and a regular surface [24,25].

So far, there are single reports on the use of HFUS in the assessment of AK, including the use of this imaging method in evaluating the effects of therapy. Among others, the thickness and echogenicity of SLEB have been analyzed as parameters related to the presence of atypical keratinocytes. However, it is known that inflammation or elastosis may affect the formation of this band [14,24,25]. Therefore, this study aimed to examine the usefulness of HFUS in the assessment of AK using machine learning-based feature extraction analysis and to show how skin affected by AK changes as a result of the use of PDT in a 3-month assessment.

## 2. Materials and Methods

**Figure 1:**
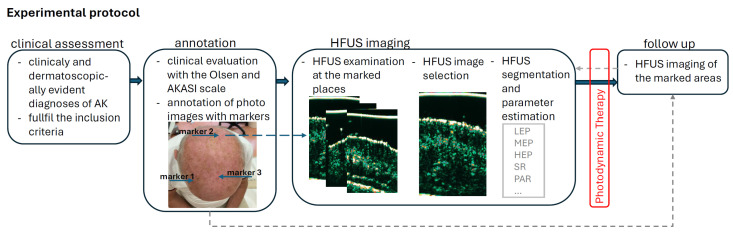
Experimental protocol.

The experimental protocol is shown in Figure 1. We included 44 AK patients aged 53 to 89 years (median age 73 years, 70% male) presenting to our Department from June 2023 to May 2024 with clinicaly and dermatoscopically evident diagnoses of AK. The lesions were on the face (27 patients) or scalp (17 patients). Patients with prior dermatological treatment, ulcerated lesions, invasion features in dermatoscopy, allergy to photosensitizers, and other chronic dermatoses in the treatment area were excluded from PDT treatment. All patients provided informed consent for the procedure. All patients underwent only one PDT session before the follow-up visits.

All patients were clinically and dermatoscopically evaluated with the Olsen [8] and AKASI scales [17], and then each lesion was marked in a photograph for follow-up visits (see Figure 1). HFUS images were acquired with a linear probe (20 MHz) and B-mode scan (Dermascan C^®^; Hadsund, Denmark). The axial and lateral resolutions were 80 and 200 µm, respectively. For each marked lesion, the dermatologist selected the HFUS image that presented the most potent manifestation of AK. If possible, in examined patients, skin scans without clinically evident AK lesions were also carried out within a close or contralateral localization to the affected region, which would serve as a reference for the healthy skin. Afterward, 5-ALA was applied on the lesional skin under occlusion for 3 h. If necessary, a curette was used to remove the scales a few days before the procedure.

After removing the occlusive dressing, 5-ALA was cleaned with a saline solution, and a directed therapy with a BF-200 lamp (narrow-emission spectrum of 635 nm ± 9 nm) was performed according to the manufacturer’s recommendations [26].

**Figure 2:**
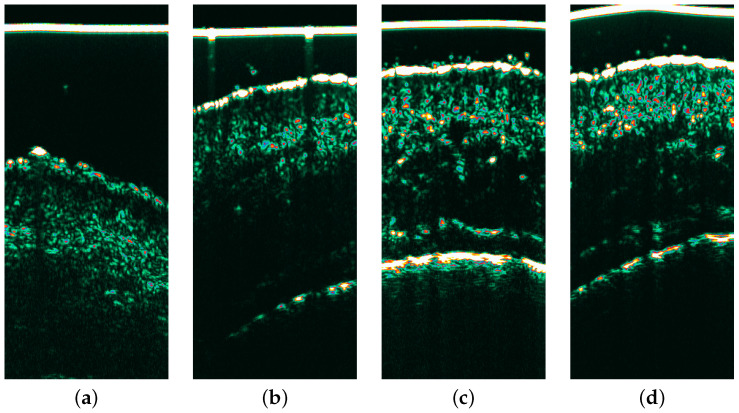
Ultrasound imaging of the AK1 site: (**a**) before therapy, (**b**) follow-up week 4, (**c**) follow-up week 8, (**d**) follow-up week 12. A reduction in the SLEB layer is visible within the 4, 8, 12 week follow-ups and the occurrence of hyperechogenic pixels throughout the patients’ visits.

**Figure 3:**
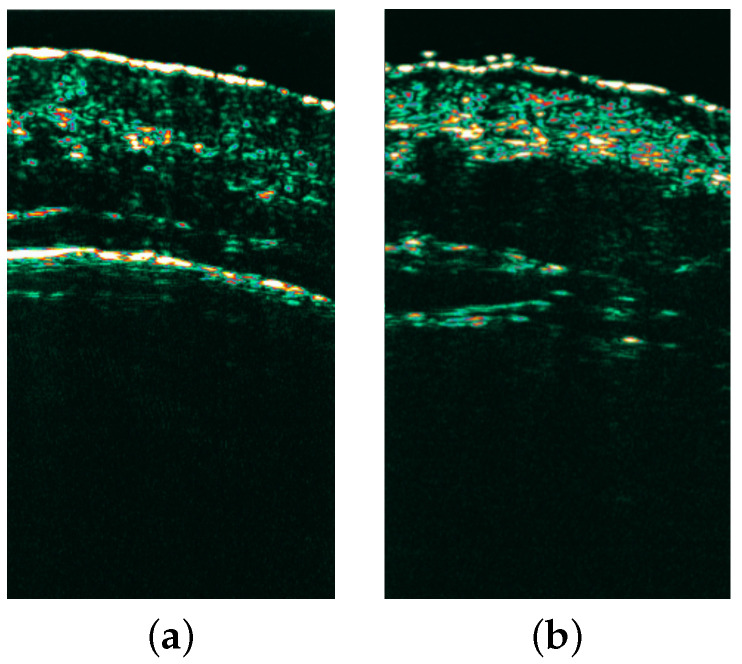
Comparison between healthy skin in a 30-year-old (**a**) and one of the evaluated patients’ clinically unaffected skin (**b**). An SLEB layer is seen in the second scan (**b**) underneath the entry echo, which might correspond to elastosis, subclinical lesions, or inflammation.

The patients were scheduled for follow-up visits 4, 8, and 12 weeks after the treatment procedure (example HFUS images shown in Figure 2). During all visits, the non-invasive procedures within marked areas were repeated. Contralateral, unaffected skin was used as a control unless it showed clinically and dermoscopically obvious signs of photodamage [27]. The control group consisted of 35 patients. A comparison between healthy skin on a 30-year-old and one of the evaluated patients’ clinically unaffected skin is shown in Figure 3.

This study was approved by the Local Ethics Committee (Poznan University of Medical Sciences, Protocol Number 523/23).

### 2.1. Feature Extraction

In [28], skin layer contours were used to estimate skin features from images using a constant thickness of layers located below the epidermal contour. However, in our study, the markers were located at different points on the face and scalp. Along with differences in the age of the patients, this can affect the thickness of the individual layers, so it was decided that the skin layers should be contoured separately for each image. Expert outlines, supported by the machine learning method, were then used to extract features for entry echo, SLEB, and dermis [28,29].

In [30,31,32,33], the authors reported the relationship between the thickness of skin layers (entry echo, SLEB, and dermis) and skin condition. Based on the contours, the thickness of each layer was determined as the average thickness measured within the layer. Variation in the thickness across the marker area was also obtained for the entry echo. The thickness variation index (TVI) was calculated as the deviation in the thickness of the entry echo layer. A low value indicates that the skin layer has a consistent thickness, while higher values suggest the presence of regions with significantly different thicknesses.

The roughness of the skin surface was described using parameters determined from the outline of the entry echo layer. The surface roughness (SR) quantifies the height variability along the layer’s upper edge. The complexity of the entry echo outline is also described by two other parameters: the ratio of the layer’s perimeter to its area and the ratio of the layer’s area to its convex hull (the smallest geometric contour of a shape). The perimeter-to-area ratio (PAR) describes the degree of irregularity in the skin layer’s outline relative to the area of the layer. A lower value suggests a smoother, more regular surface. The area-to-convex hull ratio (ACR) measures how closely the skin layer’s shape adheres to its convex hull (the smallest geometric contour of a shape). A lower value indicates a more irregular shape, whereas a value close to 1 suggests that the layer is smoother. All three parameters represent the degree of jaggedness and irregularity of the entry echo surface.

Skin echogenicity is a parameter used in the evaluation of skin aging [28,32] and the progress of therapy [30,31]. As proposed in [30], pixels were divided based on their intensity into low (<30), medium (50–150), and high echogenicity (>200). Echogenicity features were then calculated as the ratio of low-echogenic pixels (LEP), medium-echogenic pixels (MEP), and high-echogenic pixels (HEP) to all pixels within the skin layer area [28]. Echogenicity is complemented by information on the mean pixel intensity (MPI) within each layer.

In addition, the standard deviation of the pixel intensities within a given skin layer, defined as pixel intensity variability (PIV), was calculated. A higher variability means a broader range of pixel intensities, indicating more variation in brightness or texture. Complementary to this parameter is the entropy of pixel intensity (EPI), quantifying the level of disorder or randomness in the layer texture. A low value suggests that the area is more homogeneous. The following textural features were also determined using a Gray Level Co-occurrence Matrix (GLCM): correlation, homogeneity, energy, and contrast [28,34]. Correlation measures how linearly related the pixel values are within the area. A high value indicates that the pixel intensities exhibit a strong relationship, meaning that the image has well-defined and consistent patterns or textures. Homogeneity assesses how similar the pixel values are across the layer. A high homogeneity value means the pixel intensities are nearly the same throughout the region, indicating uniformity. Energy reflects the degree of uniformity or repetition in the image’s texture. High energy signifies the image’s solid and repeatable patterns, such as regular shapes or uniform regions. Contrast quantifies the difference in gray levels between neighboring pixels. A high contrast value suggests significant variations in intensity, indicating the presence of distinct textures. They provide useful information concerning the structure of the monitored regions, indicating the appearance of texture patterns or homogeneous areas.

### 2.2. Statistical Analysis

First, AK changes over successive weeks of therapy were compared. The collected data met the assumptions for the Wilcoxon Signed-Rank test, comparing the positive and negative ranks of the differences. A one-sided test was used to determine its nature if a statistically significant difference was detected. In assuming α = 0.05, test power = 0.9, and effect size = 0.5, the group size was estimated for at least 51 samples. In addition, the effect of therapy on the AKASI score of fully treated patients was examined using the Wilcoxon Signed-Rank test. All calculations were performed using G*Power 3.1.9.7. [35,36].

The results obtained after 12 weeks of therapy were then compared with healthy skin recorded in the same group of patients using the Mann–Whitney test. First, a two-sided test was used to determine whether statistical differences existed between the post-treatment values and the healthy skin. When a statistically significant difference was detected, a one-sided test was used to determine the nature of this difference. In addition, the results at week 12 of the therapy were compared according to the stage of AK using the Krusgal–Wallis and Dunn’s test. Further analysis was performed in R Studio 2022.02.3.

## 3. Results

We examined 108 AK1, 53 AK2, and 36 AK3 samples during the study. In total, 133 markers were registered at week 4, 72 at week 8, and 126 at week 12. However, full follow-up, i.e., recorded images for 4, 8, and 12 weeks, was achieved for 63 (32%) lesions. After carefully checking the quality of the recorded images, 56 (28%) markers were included in the study (see Table 2). Among the qualified markers, 34 were graded as AK1; 17, AK2; and 5, AK3. The number of healthy skin images recorded (control group) was 35, which is related to the clinically evident sun damage in the remaining patients.

### 3.1. Comparison of HFUS Skin Parameters Before Therapy and During Subsequent Follow-Up Visits

Statistical differences obtained for morphological features are presented in Table 3, and echogenicity and pixel intensity dispersion features are in Table 4. The entry echo layer and dermis thickness were significantly lower at week 8 of treatment than before (*p* = 0.0143 and *p* = 0.0001). At the same time, there was no significant difference between the thickness before and during the 12-week follow-up (see Figure 2). The thickness of SLEB significantly decreased in the following weeks compared to the pre-therapy results, reaching its lowest value after 12 weeks (*p* < 0.0001).

Variation in the thickness of the entry echo layer was significantly lower at 4, 8, and 12-week follow-up than before therapy (*p* = 0.0004, *p* < 0.0001, and *p* = 0.0014). As for the parameters describing the smoothness of the surface, ACR was significantly higher at 8 and 12 weeks compared to baseline (*p* = 0.0015 and *p* = 0.0214), and the surface roughness was significantly lower at 8-week follow-up (*p* = 0.0038).

In the entry echo layer, both the LEP and MEP ratios were significantly lower (*p* < 0.0001 and *p* < 0.001) and HEP was higher (*p* < 0.0001) in each follow-up than before therapy (see Figure 3 for more details). In the case of SLEB, the LEP ratio was significantly lower before therapy and during each follow-up (*p* = 0.0002 and *p* < 0.0001). In comparison, the MEP ratio was higher before therapy and during each follow-up (*p* ≤ 0.0001). No significant differences were recorded in the HEP ratio. In the dermis, the LEP ratio was significantly lower at 4 weeks and 12 weeks (*p* < 0.0001) compared to baseline. Both the MEP and HEP ratios were significantly higher at 4 weeks (*p* ≤ 0.0001) and 12 weeks (*p* < 0.0001 and *p* = 0.0022).

A significant increase in the mean pixel intensity across all layers was observed in 4 (*p* < 0.0001) and 12 weeks (*p* < 0.0001 for entry echo and dermis, *p* = 0.0022 for SLEB) compared to baseline. For an 8-week comparison, a statistically significant improvement was noted only for the entry echo layer (*p* < 0.0001) and SLEB (*p* = 0.0003).

In the case of the entry echo layer, pixel intensity variability decreased significantly in the following weeks of therapy (*p* = 0.0012, *p* = 0.04232, and *p* = 0.0006). While the ratios of LEP and MEP decreased and HEP increased, the layer became brighter and more uniform. There were some significant differences in the GLCM contrast (*p* < 0.001) and GLCM correlation (*p* < 0.05), which were higher in the following weeks versus before therapy.

For SLEB, the entropy of pixel intensities decreased (*p* < 0.001), and the PIV increased (*p* ≤ 0.01) compared to the pre-treatment state. GLCM contrast increased (*p* < 0.001), and two of the GLCM correlation coefficients decreased significantly (*p* < 0.05) in the following weeks compared to the baseline. The layer became even brighter, with a dominance of medium-intensity pixels.

For the dermis layer, there was a significant increase in pixel intensity variability (*p* ≤ 0.0001) and GLCM contrast (*p* < 0.001) at 4- and 12-week follow-up compared to the baseline. GLCM homogeneity decreased significantly in 4 and 12 weeks (*p* < 0.01), and GLCM energy coefficients decreased significantly between 12 weeks of observation and before treatment (*p* < 0.01). This indicates the appearance of a varied texture in the dermis layer, with an increased proportion of medium- and high-intensity pixels. Details of all calculated features are summarized in Appendix A.

### 3.2. Comparison of Skin Parameters at 12-Week Follow-Up with Healthy Skin (Control Group)

In recordings of seemingly healthy skin, the SLEB layer was present in 71% (in 25 out of 35) of markers, and in the 12-week follow-up, this layer was still visible in 88% (49 out of 56) of cases. In the entry echo layer, the MEP and LEP ratios were significantly lower, and the HEP ratio and mean pixel intensity were higher in the 12-week follow-up (*p* < 0.0001) compared to healthy skin.

In SLEB, the MEP ratio and mean pixel intensity were significantly higher in the 12-week follow-up (*p* = 0.0009 and *p* = 0.0005), but no differences for LEP and HEP were recorded. Divergence in pixel intensity, measured by the standard deviation of the intensity and GLCM coefficients, was significantly higher in the 12-week follow-up (*p* = 0.0006 and *p* < 0.05). Pixel entropy in the dermis layer was significantly higher at week 12 (*p* < 0.0001). However, there were no statistical differences between seemingly healthy skin and skin at 12-week follow-up for parameters describing echogenicity and, primarily, skin texture. A comparison of the post-treatment results obtained for three stages revealed that significant differences occurred only in two parameters related to SLEB (EPI and thickness, *p*-value < 0.002) and two related to the entry echo (GLCM correlation and PAR, *p*-value < 0.05) only between stages 1 (AK1) and 2 (AK2). When individual stages were compared with healthy skin, the results obtained for grade 3 (AK3) were most similar to those of healthy skin, while improvements in stages 1 and 2 were statistically significant. For more details, see Appendix A.

### 3.3. AKASI Score Before and After Therapy

**Figure 4:**
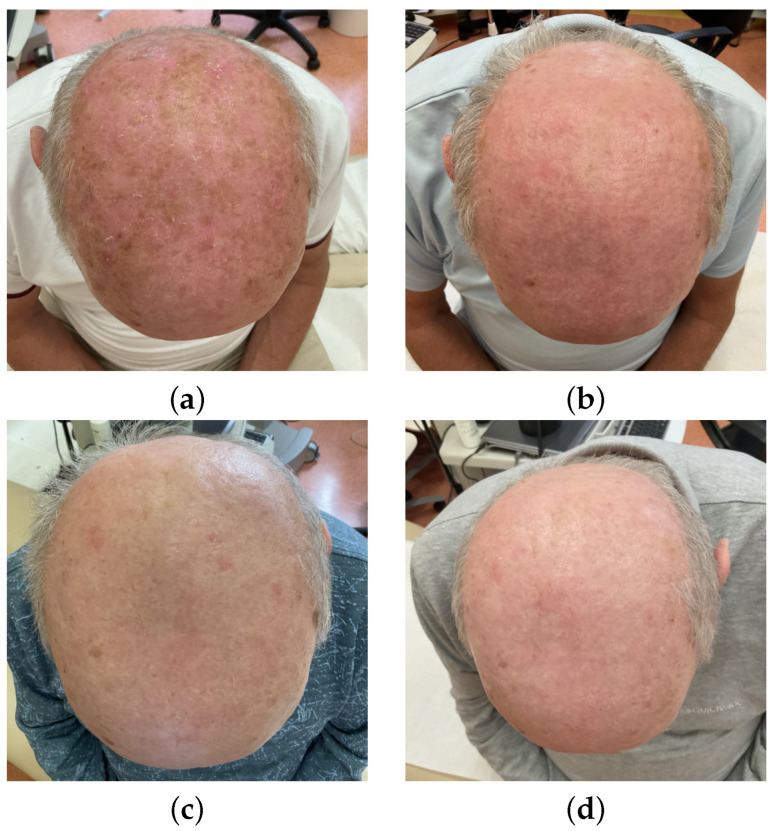
Clinical imaging: (**a**) before therapy, (**b**) follow-up at week 4, (**c**) follow-up at week 8, (**d**) follow-up at week 12.

The AKASI scores before and in 12 weeks of observation were determined for the 39 patients who underwent follow-up at week 12. The median AKASI score was 3.2 (1.2–8.6) before treatment and 0.6 (0–2.8) after therapy. The median difference before and after treatment was equal to 2.8. The difference was statistically significant with a *p*-value < 0.0001 and effect size = 0.8705, i.e., large. Complete resolution of symptoms, i.e., an AKASI score of 0, was achieved in 49% (19 of 39). Clinical images of the patient before and during PDT treatment can be seen in Figure 4.

## 4. Discussion

According to the literature data, this is the first study describing the ALA-PDT treatment efficacy on different severities of AK evaluated with HFUS. In order to observe the skin changes occurring in the AK during treatment for the first time, the authors performed follow-up visits and assessed objective ultrasound parameters such as SLEB thickness, echogenicity assessment, and the thickness of individual layers and used new variables describing changes in the skin during PDT, such as surface roughness, mean pixel intensity, and parameters describing skin texture. Furthermore, these parameters were compared with those of clinically unaffected skin.

The baseline image of AK in HFUS usually presents decreased echogenicity underneath the entry echo, with the possibility of visualizing perpendicular to the entry echo shadows corresponding to the presence of keratin on the surface. A linear SLEB might be detected. It may align with tumor formation or corresponding inflammation [14]. Histopathologically, AKs can be differentiated from field cancerization by hyperkeratosis along a concomitant parakeratosis and abnormal keratinization with a lymphocytic infiltrate, which is suspected to affect the progression of the disease [37]. Furthermore, SLEB might also represent elastosis, which histopathologically correlates with the accumulation of abnormal elastotic fibers in the upper and middle dermis [38,39]. Patients with AKs usually present with typical features for sun-exposed skin; thus, even in skin without clinically evident changes, SLEB might be visible.

MAL-PDT efficacy was observed in 26 patients with AK2 with a 50 MHz transducer. It was reported that MAL-PDT increased the dermal density and reduced the SLEB when treating targeted and perilesional skin. This reflects our observations, as the SLEB thickness decreased within the control visits at 4, 8, and 12 weeks (*p* < 0.0001) within lesional skin. However, it did not disappear completely and was still present after 12 weeks, but to a lower extent. This might correspond to the elastosis. However, we cannot unequivocally rule out the presence of infiltrate, and further study in this area is necessary (including histopathological confirmation), as well as the detailed monitoring of patients in this area—because this may be the group of patients in whom AK recurrence will be more likely than in those in whom SLEB has wholly subsided.

In our results, the skin roughness in HFUS scans also improved at the 12-week interval, which was already described clinically by Szeimies et al. [40] and Reinhold et al. [26]. The quality of the skin after BF-200 PDT was enhanced substantially. The authors reported that the number of patients without skin roughness, dryness, or scaling upgraded from 15 to 63% after the procedure [41]. PDT improves texture, wrinkling, skin coloration, and reduction in telangiectasia [41]. In our study, the entry echo layer became smoother in the subsequent months of therapy (*p* < 0.05). For parameters referring to the roughness of the skin surface, significant improvement was noted at weeks 8 and 12 (*p* < 0.05).

Apart from SLEB, other skin parameters can be analyzed in HFUS scans, especially for skin texture analyses. Crisan et al. used other parameters to evaluate the efficacy of vitamin C treatment on skin rejuvenation and the changes occurring in the skin during anti-aging therapy [30]. They used three consecutive numbers: LEP is for low-, MEP for medium-, and HEP for high-echogenic pixels in the assessed scans [28,30].

In our study, LEPs decreased significantly at all follow-up visits. This relationship was observed for all skin layers (*p* < 0.001 for entry echo, SLEB, and dermis at 4 and 12 weeks). In our study, the values were related to the total number of pixels distributed within the layers because their morphological parameters may depend on the measurement site and individual patient characteristics [42]. LEP is associated with quantifying the hydration degree within the skin, collagen degeneration or elastosis, as well as the inflammation and infiltration of a malignant tumor [28,30]. The biological effect of PDT depends on a reaction of the photosensitizer with a specific wavelength, which leads to the occurrence of molecular oxygen, and then the formation of singlet oxygen [43]. This molecule is very active and substantially causes oxidative damage and cell death [43], along with reduction in the histological features of actinic damage, decreased expression of Ki-67 and p53, and reduction in elastin thickness [44,45]. MEP and HEP quantify the levels of collagen, elastin, and microfibrils [28,30]. The MEP count increased in SLEB at the 4, 8, and 12-week follow-ups, which was expected due to the reduction in the SLEB layer at follow-up. PDT treatments increase collagen I levels, which is one of the causes of the visible clinically reversed aging [46,47], with a decrease in the elastic fibers [41]. The levels of matrix metalloproteinase-3 are also enhanced, degrading and removing old collagen fibers [46,47]. A modification within the skin proteins and the activation of skin fibroblasts can be observed [41,47], which might be visible in the MEP and HEP increases in the dermis throughout the 4-, 8-, and 12-week intervals of our patients. Since there is a quantitative change in the skin texture, PDT remains a cosmetically acceptable treatment modality that does not lead to skin atrophy or scarring compared to cryotherapy [48].

Our study additionally compared skin at a 12-week follow-up with scans obtained within clinically unchanged skin (without noticeable, visible changes associated with sun damage) in 32 treated patients. SLEB was present in most cases in both groups, but there was no statistically significant difference in the thickness of this layer. Echogenicity in this layer was comparable (LEP and HEP ratios) or better (MEP ratio and mean pixel intensity, *p* < 0.001) at week 12. At week 12, the contrast within the layer was also higher than for unchanged healthy skin (*p* < 0.05). The echogenicity of the dermis was comparable in both groups. A significant limitation to the collection of clinically unaffected skin samples in this patient group is the nature of the disease itself with the presence of field cancerization, meaning that subclinical changes might occur. The similarity of the LEP, HEP, MEP, MPI, EPI, and PIV ratios in this group might be explained by the fact that patients with photodamage tend to expose their entire skin to the sunlight. Therefore, we assume that the clinically healthy skin might comprise subclinical changes (seemingly healthy skin), which may be a topic of future studies. Without histopathological examination, we cannot be sure whether the changes that we see in this group are elastosis, inflammatory infiltrate, or other structures. The comparison between healthy skin in a young patient and a patient in our control group is featured in Figure 3.

Our results indicate that AK differentiation based on ultrasound analysis may be limited due to the small number of patients with AK3 in this study. Further studies are necessary in this area. It is worth adding that SLEB and the thickness of the entry echo seem to be a promising indicator in this area.

For the first time, in this study, we analyzed new parameters in a post-therapeutic assessment of PDT. Homogeneity corresponds to the uniformity of pixels; energy describes how texture forms into visible patterns; EPI quantifies the level of randomness within pixels; correlation shows the improvement in skin quality after therapy, corresponding to how pixels are related in the skin layers. Variations in pixel intensities are described with certain parameters such as contrast and PIV, with high values indicating the presence of distinctive patterns. These imply the improvement of skin texture and the clinically visible enhancement of skin quality.

Our study, similarly to previous ones, shows that PDT is a highly effective form of treatment that leads to reduction in AKs along with an enhancement of skin quality [46,47]. This is visible in the significant drop in our group’s AKASI score, which objectively allowed us to assess the treatment outcome [49]. We think that HFUS can be a valuable non-invasive modality in monitoring the treatment efficacy after PDT. The evolution of skin layers seen in the aforementioned parameters lets us see whether the therapy was successful or if the patients require an additional procedure.

The limitation of our study is the usage of one ultrasound machine. Further research comparing skin parameters obtained for images acquired with various devices would complement the analysis. Moreover, accurate ultrasound analysis relies on the physician’s experience in patient examination and HFUS image selection. Therefore, the analysis of multiple HFUS images acquired at the lesion area should be a part of further investigation.

Regardless, fast, non-invasive treatment monitoring might be helpful in clinical practice. However, studies focused on selected AK grades II and III might be required. HFUS devices might not be as available as dermatoscopes. Nevertheless, they allow the visualization of deeper layers of the skin, which is essential in treatment monitoring. They may enable the examination of subclinical lesions and indicate which patients may require monitoring because the risk of relapse may be higher.

## 5. Conclusions

Our study confirms that HFUS can be helpful in monitoring the effects of PDT and can complement the clinical-dermatoscopic assessment. We believe that HFUS might not only show the possible effect of PDT in decrease of SLEB but also detect subclinical lesions and allows us to analyze deeper layers of the skin.

## Figures and Tables

**Table 1:** A comparison of HFUS and traditional dermatoscopy.

	Dermatoscopy	HFUS
**Method (biophysics)**	Diagnostic technique based	It is based on the reflection
	on the Tyndall effect	of ultrasound waves
	and Rayleigh scattering	from the difference
	phenomenon	in cell structure
**Resolution/imaging**	Allows the visualization of	A 20–100 MHz transducer
**depth**	skin structures with	allows a resolution of
	polarized and non-polarized	80–200 μm. The higher
	light at 6- to 100-fold	the frequency, the lower
	magnification, reaching	the depth penetration
	down to the papillary	
	layer of the dermis	
**Limitations**	Higher number of	HFUS does not have specific
	unnecessary excisions	AK features, and reduced
	(false positive diagnosis)	echogenicity is observed in
	or false negative diagnosis	other skin cancers,
	when the tumor displays	overestimating tumor size
	features typical for	Limited specificity in the assessment
	a benign lesion	of neoplastic lesions including AK,
		possibility of overestimating tumor
		size due to inflammation and
		elastosis
**Availability**	Available for clinicians	Less frequently applied
	in every office, easy to learn	in clinical practice,
		not available in every
		dermatological office
**Costs**	Low	High

**Table 2:** Inclusion and exclusion criteria for markers.

Inclusion	Exclusion	Number of Markers After Exclusion
Flitzpatrick skin type I-II	Previous dermatological treatment, other chronic dermatoses in the examination area. Allergy to photosensitisers.	N = 197
AKs diagnosed by dermatological examination	Inability to participate in a follow-up visit.	N = 63
	Insufficient quality of any of the images in the follow-up.	N = 56

**Table 3:** Parameters describing the morphology of the skin layers on ultrasound before therapy and at weeks 4, 8, and 12 of treatment. *P*-values < 0.05 are marked in bold. Small effect size is marked as *, moderate as **, and large as ***. Thicknesses are given in [mm].

	Week 0	Week 4	*p* 0–4 Weeks	Effect Size	One-Way *p*	Week 8	*p* 0–8 Weeks	Effect Size	One-Way *p*	Week 12	*p* 0–12 Weeks	Effect Size	One-Way *p*
*Entry echo*													
Thickness	0.1981 ± 0.02	0.1937 ± 0.03	0.596	0.0714 *		0.1848 ± 0.02	**0.0285**	0.2932 *	**0.0143**	0.1951 ± 0.02	0.8035	0.0338 *	
TVI	0.0423 ± 0.01	0.0337 ± 0.01	**0.001**	0.4393 **	**0.0004**	0.0318 ± 0.01	**0.0001**	0.5853 ***	**0.0001**	0.0319 ± 0.01	**0.0033**	0.3935 **	**0.0014**
PAR	0.1716 ± 0.02	0.1687 ± 0.02	0.9707	0.0055 *		0.1731 ± 0.02	0.5597	0.0785 *		0.1637 ± 0.02	0.436	0.1046 *	
SR	11.9893 ± 6.76	10.9937 ± 5.26	0.0568	0.2551 *		8.339 ± 5.04	**0.0083**	0.3532 **	**0.0038**	9.8148 ± 3.63	0.0747	0.2387 *	
ACR	0.5084 ± 0.04	0.5503 ± 0.06	0.0695	0.2431 *		0.5804 ± 0.06	**0.0035**	0.3902 **	**0.0015**	0.5472 ± 0.06	**0.0435**	0.2703 *	**0.0214**
*SLEB*													
Thickness	0.4039 ± 0.09	0.3208 ± 0.07	**0.0001**	0.6181 ***	**0.0001**	0.3411 ± 0.08	**0.0006**	0.4611 **	**0.0003**	0.2325 ± 0.11	**0.0001**	0.7423 ***	**0.0001**
*Dermis*													
Thickness	1.5482 ± 0.21	1.469 ± 0.20	**0.0336**	0.2845 *	0.0164	1.3694 ± 0.18	**0.0003**	0.4829 **	**0.0001**	1.5124 ± 0.18	0.2946	0.1406 *	

**Table 4:** Parameters describing the echogenicity and distribution of pixels before therapy and at weeks 4, 8, and 12 of treatment. Values of *p* < 0.05 are marked in bold. Small effect size is marked as *, moderate as **, and large as ***.

	Week 0	Week 4	*p* 0–4 Weeks	Effect Size	One-Way *p*	Week 8	*p* 0–8 Weeks	Effect Size	One-Way *p*	Week 12	*p* 0–12 Weeks	Effect Size	One-Way *p*
*Entry echo*													
LEP ratio	0.0415 ± 0.03	0.0119 ± 0.01	**0.0001**	0.6028 ***	**0.0001**	0.017 ± 0.01	**0.0001**	0.5472 ***	**0.0001**	0.011 ± 0.01	**0.0001**	0.6202 ***	**0.0001**
MEP ratio	0.3991 ± 0.09	0.281 ± 0.07	**0.0011**	0.4382 **	**0.0004**	0.3196 ± 0.07	**0.0018**	0.4186 **	**0.0007**	0.2808 ± 0.07	**0.0009**	0.4426 **	**0.0004**
HEP ratio	0.3951 ± 0.17	0.6076 ± 0.11	**0.0001**	0.6191 ***	**0.0001**	0.5616 ± 0.11	**0.0001**	0.5755 ***	**0.0001**	0.6325 ± 0.1	**0.0001**	0.6039 ***	**0.0001**
MPI	158.9785 ± 23.9	193.7715 ± 18.19	**0.0001**	0.6148 ***	**0.0001**	186.7654 ± 15.88	**0.0001**	0.5843 ***	**0.0001**	199.0126 ± 13.97	**0.0001**	0.6104 ***	**0.0001**
EPI	0.1823 ± 0.02	0.1801 ± 0.02	0.6984	0.0523 *		0.1718 ± 0.01	**0.0186**	0.315 **	**0.0089**	0.1817 ± 0.02	0.6864	0.0545 *	
PIV	74.2705 ± 3.52	69.6094 ± 4.16	**0.0029**	0.399 **	**0.0012**	71.3299 ± 3.14	**0.047**	0.266 *	**0.0232**	68.1178 ± 3.71	**0.0014**	0.4262 **	**0.0006**
*SLEB*													
LEP ratio	0.7556 ± 0.12	0.5564 ± 0.16	**0.0001**	0.5341 ***	**0.0001**	0.6123 ± 0.08	**0.0006**	0.4611 **	**0.0002**	0.4247 ± 0.2	**0.0001**	0.6617 ***	**0.0001**
MEP ratio	0.1129 ± 0.06	0.2119 ± 0.1	**0.0001**	0.6061 ***	**0.0001**	0.1923 ± 0.06	**0.0001**	0.5232 ***	**0.0001**	0.258 ± 0.13	**0.0002**	0.5025 ***	**0.0001**
HEP ratio	0 ± 0	0 ± 0	0.9653	0.002 *		0 ± 0	0.4513	0.1388 *		0 ± 0	0.3279	0.1804 *	
MPI	27.069 ± 7.12	36.9444 ± 9.45	**0.0001**	0.5363 ***	**0.0001**	35.7611 ± 5.06	**0.0009**	0.4436 **	**0.0003**	41.5227 ± 12.94	**0.005**	0.3761 **	**0.0022**
EPI	0.3132 ± 0.05	0.2487 ± 0.06	**0.0001**	0.5973 ***	**0.0001**	0.2721 ± 0.06	**0.0008**	0.4491 **	**0.0003**	0.2053 ± 0.08	**0.0001**	0.7445 ***	**0.0001**
PIV	22.3682 ± 5.65	29.7001 ± 6.3	**0.0003**	0.4894 **	**0.0001**	27.7261 ± 4.56	**0.0011**	0.436 **	**0.0004**	31.1373 ± 6.6	**0.0207**	0.3096 **	**0.01**
*Dermis*													
LEP ratio	0.5957 ± 0.11	0.4622 ± 0.13	**0.0001**	0.5984 ***	**0.0001**	0.5639 ± 0.12	0.0734	0.2398 *		0.482 ± 0.13	**0.0001**	0.5537 ***	**0.0001**
MEP ratio	0.2438 ± 0.09	0.3453 ± 0.1	**0.0001**	0.5777 ***	**0.0001**	0.271 ± 0.1	0.089	0.2278 *		0.3304 ± 0.11	**0.0001**	0.5526 ***	**0.0001**
HEP ratio	0.0008 ± 0.01	0.0064 ± 0.01	**0.0001**	0.514 ***	**0.0001**	0.0016 ± 0.01	0.1484	0.1957 *		0.0046 ± 0.01	**0.005**	0.3761 **	**0.0022**
MPI	40.5087 ± 10.02	51.7453 ± 13.39	**0.0001**	0.5973 ***	**0.0001**	42.2963 ± 11.7	0.0558	0.2562 *		50.6193 ± 13.91	**0.0001**	0.5635 ***	**0.0001**
EPI	0.7542 ± 0.05	0.7352 ± 0.05	0.0507	0.2616 *		0.7035 ± 0.05	**0.0002**	0.4981 **	**0.0001**	0.7443 ± 0.05	0.2552	0.1526 *	
PIV	35.3741 ± 6.72	42.9911 ± 6.71	**0.0001**	0.581 ***	**0.0001**	38.0342 ± 6.06	0.086	0.23 *		42.7968 ± 6.08	**0.0001**	0.5407 ***	**0.0001**

## Data Availability

The data that support the findings of this study are available from the corresponding author upon reasonable request.

## Appendix A

Detailed results obtained during the statistical analysis are presented below. The determined GLCM features for the entry echo layer are summarised in Table A1, for the SLEB layer in Table A2 and for the dermis in Table A3. The characteristics for which significant statistical differences were shown between the control group and week 12 are summarised in Table A4. Table A5 presets results of Krusgall-Wallis and post-hoc Dunns tests.

**Table A1:** GLCM features describing the texture of the entry echo layer before therapy and at weeks 4, 8 and 12 of treatment. Values of *p* < 0.05 are marked in bold. Small effect size is marked as *, moderate as ** and large as ***.

GLCM	Week 0	Week 4	*p* 0–4 Weeks	Effect Size	One-Way *p*	Week 8	*p* 0–8 Weeks	Effect Size	One-Way *p*	Week 12	*p* 0–12 Weeks	Effect Size	One-way *p*
Contr. 1	106.5664 ± 31.8	121.4188 ± 25.42	0.0721	0.2409 *		125.8546 ± 30.04	0.0623	0.2496 *		122.6773 ± 24.68	0.1643	0.1864 *	
Contr. 2	307.6188 ± 146.20	476.6654 ± 162.24	**0.0009**	0.4458 **	**0.0003**	495.5858 ± 164.01	**0.0002**	0.4927 **	**<0.0001**	499.4119 ± 156.8	**0.0005**	0.4676 **	**0.0002**
Contr. 3	252.9212 ± 117.49	428.2498 ± 165.32	**0.0007**	0.4545 **	**0.0002**	484.8061 ± 151.88	**<0.0001**	0.5712 ***	**<0.0001**	432.2026 ± 120.73	**<0.0001**	0.5439 ***	**<0.0001**
Contr. 4	300.1705 ± 127.87	436.0123 ± 200.93	**0.0005**	0.4687 **	**0.0002**	509.565 ± 186.46	**<0.0001**	0.5832 ***	**<0.0001**	470.5519 ± 139.63	**0.0001**	0.5189 ***	**<0.0001**
Corr. 1	0.8275 ± 0.05	0.8694 ± 0.04	**0.0004**	0.4742 **	**0.0001**	0.8742 ± 0.04	**0.0003**	0.4818 **	**0.0001**	0.885 ± 0.04	**0.0048**	0.3772 **	**0.0021**
Corr. 2	0.5304 ± 0.06	0.5989 ± 0.06	**0.0246**	0.3009 **	**0.0119**	0.5563 ± 0.07	0.2451	0.1559 *		0.5997 ± 0.06	**0.0323**	0.2867 *	**0.0158**
Corr. 3	0.6188 ± 0.05	0.6273 ± 0.06	0.5543	0.0796 *		0.5924 ± 0.05	0.1202	0.2082 *		0.6242 ± 0.04	0.6804	0.0556 *	
Corr. 4	0.6065 ± 0.09	0.5722 ± 0.08	0.6274	0.0654 *		0.5236 ± 0.07	0.0953	0.2235 *		0.5811 ± 0.06	0.8098	0.0327 *	
Energy 1	0.5521 ± 0.11	0.5173 ± 0.10	0.1108	0.2136 *		0.4461 ± 0.12	**0.0156**	0.3237 **	**0.0074**	0.481 ± 0.07	**0.0479**	0.2649 *	**0.0237**
Energy 2	0.4751 ± 0.14	0.4166 ± 0.13	0.1072	0.2158 *		0.3461 ± 0.16	**0.0119**	0.3368 **	**0.0055**	0.3816 ± 0.1	0.0816	0.2333 *	
Energy 3	0.4834 ± 0.15	0.4198 ± 0.13	0.086	0.23 *		0.3383 ± 0.15	**0.0085**	0.3521 **	**0.0039**	0.3893 ± 0.1	0.0537	0.2583 *	
Energy 4	0.4852 ± 0.14	0.4161 ± 0.13	0.0937	0.2245 *		0.3248 ± 0.15	**0.0053**	0.3728 **	**0.0024**	0.3854 ± 0.09	**0.0285**	0.2932 *	**0.0139**
Homo. 1	0.8222 ± 0.04	0.84 ± 0.04	0.538	0.0828 *		0.8239 ± 0.05	0.6625	0.0589 *		0.828 ± 0.03	0.3503	0.1254 *	
Homo. 2	0.7344 ± 0.08	0.742 ± 0.07	0.4217	0.1079 *		0.6578 ± 0.1	**0.0357**	0.2812 *	**0.0175**	0.7174 ± 0.06	0.4554	0.1003 *	
Homo. 3	0.7528 ± 0.08	0.7403 ± 0.07	0.306	0.1373 *		0.6709 ± 0.11	**0.0194**	0.3128 **	**0.0093**	0.7178 ± 0.05	0.2871	0.1428 *	
Homo. 4	0.7545 ± 0.07	0.7349 ± 0.09	0.2017	0.1711 *		0.6711 ± 0.11	**0.0111**	0.3401 **	**0.0051**	0.7109 ± 0.06	0.1744	0.182 *	

**Table A2:** GLCM features describing the texture of SLEB before therapy and at weeks 4, 8 and 12 of treatment. Values of *p* < 0.05 are marked in bold. Small effect size is marked as *, moderate as **.

GLCM	Week 0	Week 4	*p* 0–4 Weeks	Effect Size	One-Way *p*	Week 8	*p* 0–8 Weeks	Effect Size	One-Way *p*	Week 12	*p* 0–12 Weeks	Effect Size	One-Way *p*
Contr. 1	45.1717 ± 16.03	71.0067 ± 35.1	**0.001**	0.4393 **	**0.0004**	67.0024 ± 20.43	**0.0045**	0.3804 **	**0.002**	86.7034 ± 47.24	**0.0014**	0.4273 **	**0.0006**
Contr. 2	60.7874 ± 29.33	103.3784 ± 59.6	**0.0009**	0.4426 **	**0.0004**	99.8159 ± 39.14	**0.0018**	0.4175 **	**0.0007**	121.2807 ± 81.58	**0.0028**	0.4 **	**0.0012**
Contr. 3	61.0687 ± 24.64	99.9934 ± 62.47	**0.0004**	0.4774 **	**0.0001**	93.4511 ± 41.82	**0.0008**	0.448 **	**0.0003**	124.7702 ± 76.91	**0.0009**	0.4436 **	**0.0003**
Contr. 4	67.3266 ± 26.37	107.7238 ± 65.68	**0.0009**	0.4436 **	**0.0003**	102.0267 ± 45.02	**0.001**	0.4393 **	**0.0004**	136.0699 ± 73.51	**0.0007**	0.4556 **	**0.0002**
Corr. 1	0.5398 ± 0.05	0.5619 ± 0.04	0.4804	0.0948 *		0.5682 ± 0.04	0.1476	0.194 *		0.565 ± 0.06	0.9577	0.0076 *	
Corr. 2	0.3428 ± 0.07	0.3373 ± 0.09	0.4855	0.0937 *		0.3373 ± 0.06	0.5931	0.0719 *		0.3579 ± 0.1	0.2227	0.1635 *	
Corr. 3	0.4007 ± 0.07	0.3678 ± 0.1	**0.0418**	0.2725 *	**0.0206**	0.3821 ± 0.08	0.0905	0.2267 *		0.3569 ± 0.11	**0.0139**	0.3292 **	**0.0066**
Corr. 4	0.3883 ± 0.07	0.327 ± 0.1	**0.0479**	0.2649 *	**0.0237**	0.3208 ± 0.07	**0.047**	0.266 *	**0.0232**	0.3252 ± 0.09	**0.0036**	0.3891 **	**0.0016**
Energy 1	0.3113 ± 0.13	0.3476 ± 0.13	0.8225	0.0305 *		0.3025 ± 0.11	0.9642	0.0065 *		0.3098 ± 0.15	0.9317	0.012 *	
Energy 2	0.2386 ± 0.13	0.2671 ± 0.14	0.9253	0.0131 *		0.2403 ± 0.11	0.6804	0.0556 *		0.2434 ± 0.16	0.6332	0.0643 *	
Energy 3	0.247 ± 0.13	0.2736 ± 0.14	0.88	0.0207 *		0.242 ± 0.11	0.6217	0.0665 *		0.2463 ± 0.16	0.5062	0.0894 *	
Energy 4	0.2498 ± 0.13	0.2749 ± 0.14	0.8035	0.0338 *		0.2403 ± 0.11	0.5488	0.0807 *		0.2444 ± 0.15	0.385	0.1166 *	
Homo. 1	0.7005 ± 0.09	0.6929 ± 0.08	0.4604	0.0992 *		0.6868 ± 0.07	0.4312	0.1057 *		0.6874 ± 0.1	0.083	0.2322 *	
Homo. 2	0.6363 ± 0.10	0.6238 ± 0.1	0.3216	0.133 *		0.6205 ± 0.08	0.2726	0.1472 *		0.6065 ± 0.12	**0.0418**	0.2725 *	**0.0206**
Homo. 3	0.6603 ± 0.09	0.6407 ± 0.1	0.2258	0.1624 *		0.6394 ± 0.08	0.2165	0.1657 *		0.6224 ± 0.12	**0.019**	0.3139 **	**0.0091**
Homo. 4	0.6439 ± 0.1	0.6348 ± 0.1	0.2258	0.1624 *		0.6233 ± 0.08	0.2017	0.1711 *		0.6074 ± 0.12	**0.0199**	0.3118 **	**0.0095**

**Table A3:** GLCM features describing the texture of dermis layer before therapy and at weeks 4, 8 and 12 of treatment. Values of *p* < 0.05 are marked in bold. Small effect size is marked as *, moderate as ** and large as ***.

GLCM	Week 0	Week 4	*p* 0–4 Weeks	Effect Size	One-Way *p*	Week 8	*p* 0–8 Weeks	Effect Size	One-Way *p*	Week 12	*p* 0–12 Weeks	Effect Size	One-Way *p*
Contr. 1	78.5713 ± 24.07	112.9283 ± 34.53	**0.0003**	0.4807 **	**0.0001**	86.2491 ± 22.28	0.0601	0.2518 *		107.6226 ± 26.38	**0.0001**	0.5243 ***	**<0.0001**
Contr. 2	94.3037 ± 31.44	137.602 ± 41.77	**0.0006**	0.4567 **	**0.0002**	109.845 ± 32.72	0.0747	0.2387 *		132.783 ± 34.58	**0.0003**	0.4818 **	**0.0001**
Contr. 3	78.4161 ± 25.86	107.8711 ± 36.84	**0.0036**	0.3891 **	**0.0016**	88.7475 ± 27.92	0.1108	0.2136 *		108.7588 ± 30.2	**0.0011**	0.4382 **	**0.0004**
Contr. 4	93.71 ± 29.95	132.7353 ± 44.95	**0.0004**	0.4774 **	**0.0001**	106.4666 ± 29.61	0.0537	0.2583 *		130.9523 ± 34.96	**0.0001**	0.5145 ***	**<0.0001**
Corr. 1	0.5657 ± 0.03	0.5775 ± 0.04	0.1409	0.1973 *		0.565 ± 0.05	0.6274	0.0654 *		0.5704 ± 0.04	0.2834	0.1439 *	
Corr. 2	0.4679 ± 0.04	0.5025 ± 0.05	0.0547	0.2572 *		0.4595 ± 0.05	0.4456	0.1025 *		0.4705 ± 0.06	0.262	0.1504 *	
Corr. 3	0.5682 ± 0.04	0.5946 ± 0.04	**0.0055**	0.3717 **	**0.0024**	0.5732 ± 0.05	0.3256	0.1319 *		0.5812 ± 0.04	**0.0139**	0.3292 **	**0.0066**
Corr. 4	0.4927 ± 0.04	0.492 ± 0.05	0.3985	0.1134 *		0.4608 ± 0.05	0.1454	0.1951 *		0.4801 ± 0.06	0.5273	0.085 *	
Energy 1	0.0988 ± 0.05	0.081 ± 0.04	0.0788	0.2354 *		0.0868 ± 0.05	0.1476	0.194 *		0.0683 ± 0.03	**0.0026**	0.4022 **	**0.0011**
Energy 2	0.0822 ± 0.05	0.0623 ± 0.04	0.076	0.2376 *		0.0676 ± 0.04	0.1202	0.2082 *		0.0506 ± 0.02	**0.0031**	0.3957 **	**0.0013**
Energy 3	0.0853 ± 0.05	0.0649 ± 0.04	0.0537	0.2583 *		0.0717 ± 0.05	0.2135	0.1668 *		0.0515 ± 0.03	**0.0027**	0.4011 **	**0.0011**
Energy 4	0.0801 ± 0.05	0.0643 ± 0.04	0.0547	0.2572 *		0.066 ± 0.04	0.0905	0.2267 *		0.0499 ± 0.03	**0.0025**	0.4044 **	**0.001**
Homo. 1	0.5203 ± 0.08	0.4772 ± 0.07	**0.0045**	0.3804 **	**0.002**	0.4947 ± 0.07	0.0845	0.2311 *		0.452 ± 0.04	**0.0004**	0.472 **	**0.0001**
Homo. 2	0.4869 ± 0.08	0.4374 ± 0.08	**0.0035**	0.3913 **	**0.0015**	0.446 ± 0.08	**0.0418**	0.2725 *	**0.0206**	0.4074 ± 0.05	**0.0005**	0.4644 **	**0.0002**
Homo. 3	0.5063 ± 0.08	0.4549 ± 0.07	**0.0039**	0.3859 **	**0.0017**	0.4684 ± 0.08	0.1643	0.1864 *		0.4246 ± 0.05	**0.001**	0.4404 **	**0.0004**
Homo. 4	0.4923 ± 0.08	0.4412 ± 0.08	**0.0019**	0.4164 **	**0.0008**	0.4501 ± 0.08	**0.0336**	0.2845 *	**0.0164**	0.406 ± 0.05	**0.0003**	0.4785 **	**0.0001**

**Table A4:** Features that are statistically different (*p* < 0.05) for seemingly healthy and 12 weeks skin. Small effect size is marked as *, moderate as ** and large as ***.

	Healthy	Week 12	*p* Two-Way	Effect Size	*p* One-Way
*Entry echo*					
LEP ratio	0.0458 ± 0.03	0.011 ± 0.01	0.0001	0.5388 ***	0.0001
MEP ratio	0.4396 ± 0.08	0.2808 ± 0.07	0.0001	0.5191 ***	0.0001
HEP ratio	0.3649 ± 0.12	0.6325 ± 0.1	0.0001	0.5841 ***	0.0001
EPI	0.1185 ± 0.02	0.1817 ± 0.02	0.0001	0.8321 ***	0.0001
MPI	151.2504 ± 21.22	199.0126 ± 13.97	0.0001	0.5465 ***	0.0001
PIV	73.5075 ± 3.51	68.1178 ± 3.71	0.0016	0.3309 **	0.0008
Contr. 2	702.1389 ± 296.76	499.4119 ± 156.8	0.0364	0.2198 *	0.0182
Contr. 3	641.9425 ± 289.07	432.2026 ± 120.73	0.0051	0.2942 *	0.0025
Contr. 4	628.9161 ± 300.47	470.5519 ± 139.63	0.0116	0.2651 *	0.0058
Corr. 1	0.853 ± 0.03	0.885 ± 0.04	0.0194	0.2454 *	0.0097
Corr. 2	0.1909 ± 0.11	0.5997 ± 0.06	0.0001	0.7953 ***	0.0001
Corr. 3	0.1748 ± 0.09	0.6242 ± 0.04	0.0001	0.8329 ***	0.0001
Corr. 4	0.1084 ± 0.1	0.5811 ± 0.06	0.0001	0.785 ***	0.0001
Homo. 1	0.7987 ± 0.08	0.828 ± 0.03	0.0085	0.2762 *	0.0043
Homo. 2	0.6046 ± 0.15	0.7174 ± 0.06	0.002	0.325 **	0.001
Homo. 3	0.6071 ± 0.15	0.7178 ± 0.05	0.0007	0.3566 **	0.0003
Homo. 4	0.5923 ± 0.15	0.7109 ± 0.06	0.0007	0.3566 **	0.0003
Thickness	0.1141 ± 0.02	0.1951 ± 0.02	0.0001	0.8338 ***	0.0001
TVI	0.0215 ± 0.00	0.0319 ± 0.01	0.0001	0.5268 ***	0.0001
PAR	0.2638 ± 0.04	0.1637 ± 0.02	0.0001	0.7996 ***	0.0001
SR	6.0213 ± 2.82	9.8148 ± 3.63	0.0004	0.3737 **	0.0002
*SLEB*					
MEP ratio	0.1242 ± 0.12	0.258 ± 0.13	0.0017	0.3286 **	0.0009
MPI	28.4545 ± 19.26	41.5227 ± 12.94	0.0009	0.3483 **	0.0005
PIV	22.1524 ± 14.17	31.1373 ± 6.6	0.0012	0.3406 **	0.0006
Contr. 1	49.0758 ± 40.63	86.7034 ± 47.24	0.0015	0.3325 **	0.0008
Contr. 2	71.5932 ± 60.81	121.2807 ± 81.58	0.0038	0.3041 **	0.0019
Contr. 3	69.86 ± 61.81	124.7702 ± 76.91	0.0046	0.2972 *	0.0023
Contr. 4	67.4893 ± 65.52	136.0699 ± 73.51	0.0039	0.3033 **	0.0019
Corr. 1	0.4908 ± 0.27	0.565 ± 0.06	0.0004	0.3703 **	0.0002
Corr. 2	0.2204 ± 0.17	0.3579 ± 0.1	0.0023	0.3196 **	0.0012
Corr. 3	0.2332 ± 0.16	0.3569 ± 0.11	0.0005	0.3634 **	0.0003
Corr. 4	0.21 ± 0.13	0.3252 ± 0.09	0.0003	0.384 **	0.0001
Energy 3	0.1268 ± 0.18	0.2463 ± 0.16	0.0385	0.2173 *	0.0193
Energy 4	0.1191 ± 0.17	0.2444 ± 0.15	0.037	0.2191 *	0.0185
Homo. 2	0.4599 ± 0.32	0.6065 ± 0.12	0.0409	0.2148 *	0.0204
Homo. 3	0.4723 ± 0.32	0.6224 ± 0.12	0.0235	0.238 *	0.0117
Homo. 4	0.4553 ± 0.31	0.6074 ± 0.12	0.025	0.2354 *	0.0125
Thickness	0.1931 ± 0.16	0.2325 ± 0.11	0.0317	0.2256 *	0.0158
*Dermis*					
EPI	0.5782 ± 0.06	0.7443 ± 0.05	0.0001	0.6499 ***	0.0001
Contr. 1	77.9284 ± 18.53	107.6226 ± 26.38	0.0003	0.3771 **	0.0002
Corr. 1	0.6438 ± 0.06	0.5704 ± 0.04	0.0049	0.295 *	0.0025
Corr. 3	0.5045 ± 0.07	0.5812 ± 0.04	0.0001	0.4695 **	0.0001
Thickness	0.9761 ± 0.15	1.5124 ± 0.18	0.0001	0.6499 ***	0.0001

**Table A5:** Comparation of different stages of AK in 12 weeks of therapy.

Feature and Skin Layer	*p*-Value	Magnitude	*p* Stage 1–2	*p* Stage 1–3	*p* Stage 2–3
GLCM corr. for Entry Echo	0.0154	moderate	**0.0276**	0.2345	1
PAR for Entry Echo	**0.0397**	moderate	0.0486	1	0.2730
EPI for SLEB	**0.0013**	large	0.0034	0.0684	1
Thickness for SLEB	**0.0017**	large	0.0035	0.0951	1
